# Is searching full text more effective than searching abstracts?

**DOI:** 10.1186/1471-2105-10-46

**Published:** 2009-02-03

**Authors:** Jimmy Lin

**Affiliations:** 1 National Center for Biotechnology Information, National Library of Medicine, Bethesda, Maryland, USA; 2 The iSchool, University of Maryland, College Park, Maryland, USA

## Abstract

**Background:**

With the growing availability of full-text articles online, scientists and other consumers of the life sciences literature now have the ability to go beyond searching bibliographic records (title, abstract, metadata) to directly access full-text content. Motivated by this emerging trend, I posed the following question: is searching full text more effective than searching abstracts? This question is answered by comparing text retrieval algorithms on MEDLINE^® ^abstracts, full-text articles, and spans (paragraphs) within full-text articles using data from the TREC 2007 genomics track evaluation. Two retrieval models are examined: *bm25 *and the ranking algorithm implemented in the open-source Lucene search engine.

**Results:**

Experiments show that treating an entire article as an indexing unit does not consistently yield higher effectiveness compared to abstract-only search. However, retrieval based on spans, or paragraphs-sized segments of full-text articles, consistently outperforms abstract-only search. Results suggest that highest overall effectiveness may be achieved by combining evidence from spans and full articles.

**Conclusion:**

Users searching full text are more likely to find relevant articles than searching only abstracts. This finding affirms the value of full text collections for text retrieval and provides a starting point for future work in exploring algorithms that take advantage of rapidly-growing digital archives. Experimental results also highlight the need to develop distributed text retrieval algorithms, since full-text articles are significantly longer than abstracts and may require the computational resources of multiple machines in a cluster. The MapReduce programming model provides a convenient framework for organizing such computations.

## Background

The exponential growth of peer-reviewed literature and the breakdown of disciplinary boundaries heralded by genome-scale instruments have made it harder than ever for scientists to find and assimilate all the publications important to their research [1]. Thus, tools such as search engines are becoming indispensable. Motivated by the desire to develop more effective text retrieval algorithms for consumers of the life sciences literature, this study poses a straightforward question: Is searching full text more effective than searching abstracts? That is, given a particular information need expressed as a query, is the user more likely to find relevant articles searching the full text or searching only the abstracts?

This question is highly relevant today due to the growing availability of full-text articles. In the United States, the NIH Public Access Policy now requires that all research published with NIH funding be made publicly accessible. More broadly, the Open Access movement for the dissemination of scientific knowledge has gained significant traction worldwide. These trends have contributed to the accumulation of full-text articles in public archives such as PubMed Central and on the websites of Open Access journal publishers. The growth of freely-available content represents a significant opportunity for scientists, clinicians, and other users of online retrieval systems – it is now possible to go beyond searching bibliographic data (abstract, title, metadata) in sources such as MEDLINE to directly search contents of the full text. Indeed, Zweigenbaum et al. [2,3] identified analysis of full-text articles as one of the frontiers in biomedical text mining.

To be clear, I refer to full-text search as the ability to perform retrieval on the entire textual content of an article (including its title and abstract), whereas abstract search refers to retrieval based only on abstract and title (similar to what is available in PubMed^® ^today). This work focuses on content-based searching only; experiments do not take advantage of metadata (e.g., authors, journal titles, MeSH^® ^descriptors), since they would presumably be available for both full-text and abstract searches.

Not surprisingly, there isn't a straightforward answer to the question posed in this study. I describe experiments with MEDLINE abstracts, full-text articles, and spans (paragraphs) within full-text articles using data from the TREC 2007 genomics track evaluation. The experiments examined two retrieval models: *bm25 *and the ranking algorithm implemented in the open-source Lucene search engine. Results show that treating an entire article as an indexing unit does not consistently yield higher effectiveness compared to abstract search. However, retrieval based on spans, or paragraphs-sized segments of full-text articles, consistently outperforms abstract search. Results suggest that highest overall effectiveness may be achieved by combining evidence from spans and full articles. These findings affirm the value of full text collections for text retrieval and provide a starting point for future work in exploring algorithms that take advantage of full text.

One important implication of this work is the need to develop scalable text retrieval algorithms, since full-text articles are significantly longer than abstracts. In the near future, the amount of available content will likely outgrow the capabilities of individual machines, thus requiring the use of clusters for basic processing tasks. I explore this issue with Ivory, a toolkit for distributed text retrieval built on top of Hadoop, an open-source implementation of the MapReduce programming model [4].

### Related Work

Despite much optimism among researchers about the potential of full-text articles, it is not completely clear how present techniques for mining the biomedical literature, primarily developed for title, abstract, and metadata (e.g., MeSH descriptors), can be adapted to full text. For some tasks, full text does not appear to offer much beyond title and abstract. For example, consider the Medical Text Indexer (MTI), a production tool at the National Library of Medicine (NLM) that assists human indexers in assigning MeSH descriptors to MEDLINE citations based on title and abstract. Gay et al. [5] reported their experiences on extending MTI to base MeSH recommendations on full text. After much tuning and careful selection of article sections, a modest gain in effectiveness was achieved. However, the improvements were most likely "not worth the extra effort" (personal communication, Alan Aronson) and there are currently no plans to integrate full-text capabilities into the NLM article processing pipeline. Furthermore, it is unclear whether the particular treatment of article sections is generalizable beyond the relatively small set of articles examined in the study.

Other researchers have also tried to exploit information in full-text articles for various text mining tasks. For example, Yu et al. [6] used surface patterns to extract synonyms for gene and protein names, and reported higher precision on full text than on abstracts. More recently, Seki and Mostafa [7] explored an "inference network" approach to mining gene-disease associations. Taking advantage of full text yields a small gain in effectiveness (consistent with the MTI findings). However, the results were based on a small collection of articles and can only be characterized as preliminary. As with the MTI experiments, there remain questions about the generalizability of the results beyond those articles examined.

There is no doubt that a significant amount of information is contained in well-written abstracts. Journal requirements for structured abstracts assist authors in crafting highly-informative prose that covers the key aspects of their work. In the clinical domain, for example, Demner-Fushman et al. [8] showed that information in abstracts is sufficient to identify articles that are potentially useful for clinical decision support. In the biomedical domain, Yu [9] examined text associated with images in articles and concluded that sentences in the abstract suffice to summarize image content. In fact, she suggested that abstract sentences may actually be superior, since associated text in the article typically describes only experimental procedures and often does not include the findings or conclusions of an experiment. In a related study, Yu and Lee [10] discovered that 88% of figures and 85% of tables can be summarized by sentences in the abstract, and that 67% of abstract sentences correspond to images in the full-text articles. These results demonstrate that, at least for some tasks, it is unclear what additional value full-text content adds beyond title, abstract, and metadata.

Additionally, researchers have compared the term content of abstracts with that of full text. Shah et al. [11] examined a collection of articles and found that abstracts were the richest source of relevant keywords. This finding was echoed by Schuemie et al. [12], who concluded that the density of useful information is highest in the abstract, but information coverage in full text is greater than that of abstracts. However, the practicality of mining full-text articles is unclear due to increased computational requirements. Both these articles focused on characterizing texts and did not examine the impact of abstract vs. full text on a text-mining task.

There has been much work on processing full-text content, for example, the automatic extraction of gene names and protein interactions in the BioCreative evaluations [13,14]. However, that body of research differs significantly from the goals of this study in that I am primarily interested in differences between full text and abstracts, and the impact of these differences on effectiveness in text retrieval. In gene identification and related information extraction tasks, the text collection is fixed, while researchers attempt to develop effective algorithms (as determined by some standard metric such as F-measure). Specifically, the tasks are designed in a manner such that the source of the text is not a relevant factor. Experiments in this article, on the other hand, focus both on algorithms *and *data.

The problem of retrieving sub-document segments of text has previously been explored in the information retrieval literature [15-24]; the task is often called passage retrieval. Recently, there has been substantial interest in XML retrieval, which shares many of the same issues as passage retrieval since XML documents naturally support retrieval at different granularities (i.e., every XML element is a potential result). The locus of research activity resides in the INitiative for Evaluation of XML Retrieval (INEX), an annual evaluation forum that began in 2002 [25]. For several years, the evaluation used a collection of XML-encoded journal articles from the IEEE Computer Society. Most relevant to this work, the genomics "track" at the 2007 Text Retrieval Conference (TREC) has explored passage retrieval in the life sciences domain. The overview paper [26] provides an entry point into that literature (also see Section 5.1). Experiments reported in this article used the test collection from the TREC 2007 evaluation.

There is one important difference between this work and the research discussed above. Whereas I ask the question "Is searching full text more effective than searching abstracts?", previous work simply assumes that the answer is *yes*. Research papers on passage retrieval often take for granted that retrieving passages provides value to the user. The INEX evaluations implicitly assume that retrieving XML fragments is desirable (although there have been studies that explore the validity of this assumption [27,28]). Since widespread availability of full text is a relatively recent phenomenon in the life sciences, and searching abstracts has been available for decades (and shown to be reasonably effective), it is worth questioning the premise that searching full text is inherently superior. Focusing on this question, however, certainly does not diminish the contributions of previous work: to the extent that full text is shown to be valuable for text retrieval in this specific domain, findings from related work can be leveraged to inform the development of full-text retrieval algorithms for the life sciences literature.

### Comparison of Full Text and Abstracts

In comparing search effectiveness on full-text articles and abstracts, it makes sense to begin by discussing their characteristics and enumerating potential advantages and disadvantages. Such an analysis could guide the interpretation of experimental results.

Length is the most obvious difference between full-text articles and abstracts – the former provides systems with significantly more text to process. Most information retrieval algorithms today build on "bag of words" representations, where text is captured as a weighted feature vector with each term as a feature [29], or as a probability distribution over terms in the case of language modeling approaches [30,31]. These models derive from statistical properties such as term frequency and document frequency. More text could yield a more robust characterization of these statistics (for example, in the language modeling framework, longer documents require less smoothing). However, the potential downside is that full text may introduce noise. For example, an article may contain an elaborate discussion about related work that does not directly pertain to the article's main focus. Articles often contain conjectures (that turn out to be incorrect) or future work (which may or may not be fruitful lines of inquiry). In these cases, term statistics may be misleading due to the presence of "distractors" that dilute the impact of important terms. In contrast, abstracts focus on the key ideas presented in the articles and little else. Similar observations have been made with respect to collections of newswire documents (e.g., [17]). Many news stories are written about one central topic, but contain many aspects that are only loosely connected – in these cases, a global characterization of the entire document may not accurately capture what the article is "about".

Another challenge with full-text articles is the variety of formats in which they are distributed. Whereas there is a small number of formats for encoding MEDLINE abstracts, full-text collections can be found in XML, SGML, PDF, and even raw HTML (e.g., the result of Web crawls). Each format is associated with idiosyncrasies that make pre-processing a challenge. Just to give one example, there are numerous ways to encode Greek symbols (*α*, *β*, ...) – sometimes, they are directly encoded in extended character sets (e.g., Unicode); often, they are "written-out" (e.g., alpha, beta, ...); in other cases, they are encoded as HTML entities (e.g., &alpha;). The prevalence of special characters in the literature complicates seemingly simple tasks such as tokenization. The problem is further exacerbated when one tries to combine full-text collections from different sources (in different formats).

Access to full text is necessary for certain fine-grained retrieval tasks, e.g., image search [3]. Recently, there has been substantial interest in this problem [32-34], based on both image features and features derived from text associated with images, e.g., captions and sentences in which the figures are referenced. Full text is obviously essential for extracting these features. For other tasks, access to full text may be desirable, e.g., question answering. Unlike a search engine, a question answering system attempts to return a response that directly answers the user's question. Due to their specificity, users' questions sometimes may not be sufficiently addressed by abstracts – often, useful nuggets of information may be found in parts of articles that are not well reflected in the abstract (e.g., in the related work or discussion sections).

However, it is also important to take into account other considerations in information seeking. Since many users are uncomfortable reading large amounts of text on screen, they often print out journal articles first before reading them in detail. In these cases, finer-grained passage and image access capabilities are most useful in helping users decide what articles they want to read, rather than directly answering users' questions. Often, complex information needs such as those faced by biologists cannot be answered with a paragraph or an image – instead, information returned by the system must be considered in the context of the *entire *article from which the segment was extracted (for example, so that the scientist can verify the experimental procedure, consider alternative hypotheses mentioned in the discussion section, etc.). For this reason, I focus on a system's ability to identify relevant articles. This corresponds to *ad hoc *retrieval, a task that has been well studied by information retrieval researchers in large-scale evaluations such as TREC.

Based on this discussion, it is clear that there are advantages and disadvantages to full-text retrieval. However, the extent to which these various factors balance out and affect search effectiveness can only be determined empirically. The next section presents a series of experiments that explore these issues.

## Results

### Test Collection

Retrieval experiments in this article were conducted with data from the TREC 2007 genomics track evaluation [26], which used a collection of 162,259 full-text articles from Highwire Press. MEDLINE records that correspond to the full-text articles were also provided as supplementary data. To standardize system output, the organizers of the evaluation divided up the entire collection into 12.6 million "legal spans" (i.e., paragraphs) that represent the basic units of retrieval. The test collection contains 36 information needs, called "topics", and relevance judgments, which are lists of legal spans that were assessed by humans to be relevant for each topic. Despite the availability of relevance judgments at the level of spans, I focused on article-level relevance in order to support a meaningful comparison of abstract and full-text search. Section 5.1 describes this test collection in more detail.

### Retrieval Conditions

A matrix experiment was devised with two different retrieval models (in three separate implementations) and three different data conditions. The goal was to compare the effectiveness of different experimental settings, as quantified by standard retrieval metrics (see Section 2.4). The retrieval models examined were:

• The Okapi *bm25 *ranking algorithm [35,36] (described in Section 5.2), as implemented in Ivory, a toolkit for distributed text retrieval. Ivory was developed with Hadoop, an open-source implementation of the MapReduce programming model (see Section 5.3 for details).

• The ranking algorithm implemented in the open-source search engine Lucene, which represents a modified *tf.idf *retrieval model (described in Section 5.2). Due to its popularity and ease of use, Lucene provides a good baseline for comparison. This ranking algorithm was also implemented in Ivory, primarily for evaluation of efficiency and scalability (see Section 2.6).

The matrix design consisted of three different data conditions. The following indexes were built (one set of indexes for Lucene, another set of indexes for Ivory):

• Abstract index, built on the abstracts and titles of articles in the Highwire collection, taken from the MEDLINE records. Each abstract was considered a "document" for retrieval purposes.

• Article index, built on the full-text articles in the Highwire collection (which include abstracts and titles). The entire text of each article was considered a "document" for retrieval purposes.

• Span index, built on the legal spans in the Highwire collection. In this experimental condition, each of the 12.6 million spans in the collection was treated as if it were a "document".

For each cell in the matrix experiment, I performed a retrieval run with 36 queries, taken verbatim from the TREC 2007 genomics track test data (see Section 5.1 for examples). With the abstract and article indexes, retrieval results consisted of 1000 article ids in rank order. For the span index, the ranked results consisted of span ids. As a post-processing step, I applied a script to create a ranking of articles from a ranking of spans, using two different methods described by Hearst and Plaunt [17]:

• Maximum of supporting spans (max): the score for an article is computed as the maximum of scores for all spans contained in that article. Article ids were sorted by this score in descending order. This method favors articles that have a single high-scoring span.

• Sum of supporting spans (sum): the score for an article is computed as the sum of scores for all spans contained in that article. Article ids were sorted by this score in descending order. This method favors articles that have many potentially-relevant spans.

In order to ensure that the post-processing script generated a ranked list of 1000 articles (same as the abstract and article conditions), 5000 spans were retrieved initially.

### Evidence Combination

An obvious extension to the matrix experiment discussed in the previous section is to integrate evidence from multiple sources. As an initial exploration, I conducted a series of experiments that combined the results of span retrieval with either article or abstract retrieval. In both cases, runs were combined by first normalizing each set of scores and then averaging scores from the different runs. The goal of this experiment was to examine the effect of combining content representations at different granularities. Note that in principle a simple average of the scores could be replaced with weighted linear interpolation (or some other evidence combination technique), but in absence of a principled approach to determining parameters, I opted not to explore this option for fear of overtraining on limited data.

### Evaluation Metrics

For all experimental conditions, I arrived at a ranking of 1000 articles, which supported a meaningful comparison across all data conditions. To evaluate effectiveness, three different metrics were collected:

• Mean average precision (MAP), the single-point metric of effectiveness most widely accepted by the information retrieval community. The standard cutoff of 1000 hits was used.

• Precision at 20 (P20), the fraction of articles in the top twenty results that are relevant. The cutoff of twenty equals the number of hits on a result page in the present PubMed interface. In a Web environment, many searchers only focus on the first page of results.

• Interpolated precision at recall of 50% (IP@R50), which attempts to capture the experience of a dedicated, recall-oriented searcher (e.g., scientist conducting a literature search). This metric quantifies the amount of irrelevant material a user must tolerate in order to find half of the relevant articles – the higher the IP@R50, the less "junk" a user must sort through. In this study, I characterize IP@R50 as a "recall-oriented" metric. The recall level of 50% was arbitrarily selected.

Section 2.5 focuses on retrieval effectiveness with Ivory, comparing *bm25 *and the implementation of Lucene's ranking algorithm. Section 2.6 focuses on efficiency, comparing Lucene with the implementation of its ranking algorithm in Ivory.

### Retrieval Effectiveness

Results of the matrix experiment described in Section 2.2 with Ivory are presented in Table 1. The three parts of the table show effectiveness in terms of MAP, P20, and IP@R50. The columns report figures for the two retrieval models: *bm25 *and the Ivory implementation of Lucene's ranking algorithm. Abstract retrieval (i.e., retrieval using the abstract index) is taken as the baseline, and the table provides relative differences with respect to this condition. The Wilcoxon signed-rank test was applied in all cases to assess the statistical significance of the results.

**Table 1:** Effectiveness of *bm25 *and the Lucene ranking algorithm on abstracts, full-text articles, and spans from full text.

**MAP**		
	Ivory (*bm25*)	Ivory (*Lucene*)

Abstract	0.163	0.129
Article	0.146 (-11%)°	0.235 (+82%)**
Span (max)	0.240 (+47%)**	0.206 (+60%)**
Span (sum)	0.192 (+18%)*	0.198 (+54%)**

		
**P20**		

	Ivory (*bm25*)	Ivory (*Lucene*)

Abstract	0.322	0.293
Article	0.158 (-51%)**	0.353 (+20%)*
Span (max)	0.357 (+11%)°	0.332 (+13%)°
Span (sum)	0.314 (-3%)°	0.317 (+8%)*

		
**IP@R50**		

	Ivory (*bm25*)	Ivory (*Lucene*)

Abstract	0.110	0.090
Article	0.163 (+48%)°	0.222 (+146%)**
Span (max)	0.212 (+93%)**	0.189 (+109%)**
Span (sum)	0.149 (+36%)*	0.159 (+77%)**

For all metrics, relative improvements over baseline are shown; ** = statistically significant (*p *< 0.01); * = statistically significant (*p *< 0.05); ° = not significant.

Results show that for abstract retrieval, *bm25 *is significantly more effective in terms of MAP than the Lucene ranking algorithm (*p *< 0.01). Comparing abstract retrieval with article retrieval (i.e., retrieval using the article index), MAP is significantly higher (*p *< 0.01) for Lucene but differences are not statistically significant for *bm25*. For the Lucene ranking algorithm, article retrieval significantly outperforms abstract retrieval for the other two metrics as well. On the other hand, for *bm25*, article retrieval either hurts (P20), or doesn't have a significant impact (IP@P50). Article retrieval, which simply treats full-text articles as longer "documents", does not appear to yield consistent gains in effectiveness.

For retrieval using the span index, the "max" strategy for generating article rankings appears to be more effective than the "sum" strategy. In terms of MAP and IP@R50, both span retrieval strategies significantly outperform retrieval with the abstract index, for both *bm25 *and the Lucene ranking algorithm. For IP@R50, in particular, the gains are quite substantial. However, span retrieval does not appear to have a significant impact on P20 compared to the abstract retrieval baseline.

Table 2 shows the results of significance testing between article retrieval and span retrieval with the "max" strategy (the more effective, and thus more interesting, of the two strategies). For *bm25*, article retrieval is significantly worse in terms of MAP and P20. None of the differences are significant for the Lucene ranking algorithm. Note that for these comparisons, there are often large differences in per-topic scores, but in many cases one run does not consistently outperform another – for this reason, substantial differences in the mean may not necessarily yield statistical significance. These results suggest that span retrieval is at least as effective as treating the entire article as an indexing unit, and at least in a few cases, span retrieval is superior.

**Table 2:** Results of significance testing comparing article retrieval with span retrieval ("max" strategy).

	Ivory (*bm25*)	Ivory (*Lucene*)
MAP	*p *< 0.01	*n.s.*
P20	*p *< 0.01	*n.s.*
IP@R50	*n.s.*	*n.s.*

Results for the evidence combination experiments are shown in Table 3, where span retrieval is combined with abstract and article retrieval. In the interest of space, effectiveness metrics are only shown for the "max" strategy. Relative differences are shown with respect to the span retrieval baseline. As with before, the Wilcoxon signed-rank test was applied in all cases to assess the statistical significance of the results. For the Lucene ranking algorithm, combining span retrieval with article retrieval yields significant gains for all three metrics. Other differences are not statistically significant.

**Table 3:** Effectiveness of *bm25 *and the Lucene ranking algorithm combining evidence from spans with evidence from abstracts and articles.

**MAP**		
	Ivory (*bm25*)	Ivory (*Lucene*)

Span (max)	0.240	0.206
Span (max) + Abstract	0.257 (+7%)°	0.216 (+5%)°
Span (max) + Article	0.257 (+7%)°	0.262 (+27%)**

		
**P20**		

	Ivory (*bm25*)	Ivory (*Lucene*)

Span (max)	0.357	0.332
Span (max) + Abstract	0.382 (+7%)°	0.349 (+5%)°
Span (max) + Article	0.343 (-4%)°	0.404 (+22%)**

		
**IP@R50**		

	Ivory (*bm25*)	Ivory (*Lucene*)

Span (max)	0.212	0.189
Span (max) + Abstract	0.215 (+1%)°	0.190 (+1%)°
Span (max) + Article	0.257 (+21%)°	0.244 (+29%)**

For all metrics, relative improvements over baseline are shown; ** = statistically significant (*p *< 0.01); * = statistically significant (*p *< 0.05); ° = not significant.

Table 4 attempts to summarize findings from all these experiments by establishing a partial rank order of different experimental conditions (based on significance testing), in terms of each effectiveness metric and retrieval model. The "sum" strategy for span retrieval is not considered since the alternative "max" strategy appears to be more effective. In all cases, integrating span-level evidence with article-level evidence either yields the highest effectiveness or is not significantly worse than the condition that yields the highest effectiveness. This suggests that combining article content and span-level analysis is an effective approach to exploiting full text in the life sciences.

**Table 4:** Comparison of different experimental conditions for *bm25 *and the Lucene ranking algorithm.

Model	Metric	Comparison
*bm25*	MAP	Span (max) + Article, Span (max) >> Abstract, Article
	P20	Span (max) + Article, Span (max), Abstract >> Article
	IP@R50	Span (max) + Article >> Abstract, Article; Span (max) >> Abstract

Lucene	MAP	Span (max) + Article >> Span (max), Article >> Abstract
	P20	Span (max) + Article > Span (max), Article; Article > Abstract
	IP@R50	Span (max) + Article > Span (max), Article >> Abstract

*A *>> *B *indicates that *A *is significantly better than *B *(*p *< 0.01); *A *> *B *indicates that *A *is significantly better than *B *(*p *< 0.05);

### Retrieval Efficiency

Given that there is value in searching full text, the question of efficiency must be addressed. The potential effectiveness gains of full text come at a significant cost in terms of dataset size. In its compressed form as distributed, the collection of 162,259 full-text articles from Highwire Press occupies 3.28 GB (12.6 GB uncompressed). The corresponding MEDLINE abstracts, also in compressed form, take up only 139 MB (502 MB uncompressed). The difference in size is more than an order of magnitude – which means that algorithms must process significantly more data to realize the potential gains of full-text content. Articles in the Highwire collection contain on average 4148 terms (after stopword removal), while abstracts contain on average only 142 terms. For reference, spans average 66 terms, or a bit less than half the length of abstracts.

Open-source text retrieval systems available today, designed to run on individual machines, would have no difficulty handling the Highwire collection and even collections that are much larger. However, these articles represent only a small fraction of the material already available or soon to be available. Based on recent estimates, records are added to MEDLINE at a rate of approximately 65 k per month. A lower bound on the growth of available full-text content can be estimated by examining the growth of PubMed Central. Over the past two years, the digital archive has grown by approximately 40 k articles per month. However, the growth rate is uneven due to retrospective conversion; more recently, this figure is closer to 20 k articles per month. Nevertheless, full-text collections will inevitably outgrow the capabilities of individual machines – the only practical recourse is to distribute computations across multiple machines in a cluster.

I have been exploring the requirements of scaling to larger datasets with Ivory, a toolkit for distributed text retrieval implemented in Java using Hadoop, which is an open-source implementation of the MapReduce framework [4]. Section 5.3 describes Ivory in more detail, but an evaluation of its efficiency is presented here. Specifically, I compare the original implementation of Lucene with the Ivory implementation of the Lucene ranking algorithm (to factor out the effects of different retrieval algorithms).

All experiments were conducted with Amazon's Elastic Compute Cloud (EC2) service, which allows one to dynamically provision clusters of different sizes. EC2 is an example of a "utility computing" service, where anyone can "rent" computing cycles at a reasonable cost. For this work, EC2 provided a homogeneous computing environment that supports easy comparison of different cluster configurations. The basic unit of computing resource in EC2 is the small instance-hour, the virtualized equivalent of a processor core with 1.7 GB of memory, running for an hour. I experimented with the following configurations:

• Lucene (version 2.0), running on a single EC2 instance. Default settings "out of the box" were used for all experiments.

• Ivory (with Hadoop version 0.17.0), running on an EC2 cluster with 10 slave instances (plus 1 instance for the master). This is comparable to a cluster with 10 cores.

• Same as above, except with 20 slave instances, comparable to a cluster with 20 cores.

As an aside, note that physical equivalents of these clusters are quite modest by today's standards. Quad-core processors are widely available in server-class machines, and with dual processor packages, a 20-core cluster is within the means of most research groups.

Running times for index construction for the three different configurations are shown in Table 5. Lucene, which was not designed to run on a cluster, takes over a day to build the span index (containing 12.6 million spans). With either cluster configurations, indexing takes less than an hour. These figures should be interpreted with the caveat that Lucene builds a richer index that supports complex query operators and that I am comparing a single core to clusters. However, the point is that Lucene cannot be easily adapted to run on multiple machines and thus indexing speed is fundamentally bound by the disk bandwidth of one machine. A cluster can take advantage of the aggregate disk bandwidth of many machines, and MapReduce provides a convenient model for organizing these disk operations (see Section 5.3).

**Table 5:** Time required for index construction, comparing Lucene to different Ivory configurations.

	Lucene (1 core)	Ivory (10 cores)	Ivory (20 cores)
Abstract	1 h 00 m 58 s	1 m 32 s	1 m 07 s
Article	19 h 09 m 23 s	17 m 21 s	9 m 57 s
Span	27 h 10 m 46 s	39 m 58 s	24 m 56 s

The speedup demonstrated by Ivory is important because time for inverted index construction places an upper bound on how fast a researcher can explore the solution space for algorithms that require manipulating the index. Since research in information retrieval is fundamentally empirical in nature, progress is driven by iterative experimentation. Thus, exceedingly long experimental cycles represent a potential impediment to advances in the state of the art.

In terms of retrieval, running times on the entire set of 36 topics from the TREC 2007 genomics track are shown in Table 6. The gains in efficiency are not quite as dramatic, but still substantial. In its present implementation, Ivory was designed for batch-style experiments, not real-time retrieval (see Section 5.3 for more discussion). These numbers are therefore only presented for reference, and should not be taken as indicative of efficiency in operational settings (where techniques such as caching can greatly reduce retrieval latency). My experiments primarily focus on indexing efficiency, which is more important for the issues explored in this study.

**Table 6:** Time required for retrieval runs, comparing Lucene to different Ivory configurations.

	Lucene (1 core)	Ivory (10 cores)	Ivory (20 cores)
Abstract (1000 hits)	1 m 42 s	51 s	40 s
Article (1000 hits)	7 m 00 s	1 m 51 s	1 m 09 s
Span (5000 hits)	21 m 32 s	11 m 57 s	8 m 25 s

Taking advantage of full-text content requires more computational resources to cope with the increased quantities of data. Inevitably, full-text collections will outgrow retrieval systems designed to run on single machines – necessitating the development of distributed algorithms. The MapReduce framework provides a practical solution for distributed text retrieval.

## Discussion

Is searching full text more effective than searching abstracts? The answer appears to be *yes*. Furthermore, experimental results suggest that span-level analysis provides a promising strategy for taking advantage of full-text content. Whereas simply treating entire articles as indexing units yields mixed results, span retrieval consistently outperforms abstract retrieval. Combining span- and article-level evidence yields the highest effectiveness across a range of experimental conditions.

Why does span retrieval work? Further analysis of results in Section 2.5 reveals some interesting observations. Focusing on the "max" strategy, Table 1 shows that, overall, span retrieval has a relatively small effect on precision (seen in the P20 scores), but a large impact on recall (seen in the IP@R50 scores). This makes sense: key ideas in an article are likely reinforced multiple times, often in slightly different ways. This potentially alleviates mismatches between query terms and terms used by authors – in essence, span indexing gives a retrieval algorithm multiple opportunities to identify a relevant article. This enhanced recall leads to higher overall effectiveness in terms of MAP.

In general, the "max" strategy for generating article rankings from span rankings appears to be more effective than the "sum" strategy. Why is this so? One possibility is the issue of length normalization. In the current implementation, longer articles tend to have higher scores simply because they contain more spans; thus, there is an inherent bias in the "sum" strategy. Length normalization plays an important role in text retrieval [37,38], but I leave a thorough exploration of this issue for future work.

The findings in this article pave the way for future advances in full-text retrieval algorithms for the life sciences, which can draw from a wealth of previous work in the information retrieval literature on passage retrieval, XML retrieval, etc. In fact, the effectiveness of span retrieval confirms a well-known finding: ranking algorithms benefit from techniques that exploit document structure, particularly for longer documents.

Remaining focused on the problem of using full-text content to improve article ranking, how in general can article structure be exploited? Within the space of "bag of words" models, strategies can be organized in terms of two questions:

• At what levels of granularity should retrieval algorithms build representations of full-text content?

• How should evidence from multiple representations be combined to rank articles?

These two questions provide context for future work. As a start, I have experimented with two different indexing granularities (full articles and spans), but alternative approaches include sliding windows [18,20], multi-paragraph segments [19], hierarchically-overlapping segments [38,39], and segments based on topic shifts [17]. There are many strategies for integrating evidence from multiple content representations and representations at different granularities (e.g., [40]). I have begun to examine some of these strategies, but there are many more possibilities yet to be explored. For example, differential treatment of article sections may improve effectiveness since some sections are more important than others, i.e., more likely to contain relevant information. Earlier work on a smaller collection of documents from the Federal Register illustrated the potential of assigning weights to different section types [19]. More recently, Tbahriti et al. [41] found section-specific weights to be helpful for retrieval in the context of structured abstracts in the life sciences. However, one challenge that must be overcome for this strategy to work on a large scale is the lack of standardized section headings – both across journals and different types of articles (e.g., research vs. review articles).

In this work I have focused on exploiting full-text content to better rank articles. Alternatively, one could leverage full text to directly return relevant information, i.e., with passage retrieval techniques. This was, in fact, the original design of the TREC 2007 genomics track evaluation. Of course, this begs the question: How are they related? In the information retrieval literature, a distinction is made between passage retrieval and document retrieval that exploits passage-level evidence. This exactly parallels the present discussion about retrieving segments of full-text content versus leveraging full-text content to enhance article retrieval. However, I argue that the two are complementary from a user interface point of view.

Leaving aside non-traditional search interfaces, a retrieval system must ultimately present users with lists of results. Consider the two approaches to exploiting full text in this context:

Even if the primary goal of a system is to leverage full-text content to enhance article retrieval, results have to be presented in a manner that suggests the relevance of an article. This necessarily involves creating some type of surrogate for the article, which can either be indicative or informative. Common techniques for generating such surrogates include displaying titles and metadata (as with the current PubMed interface) and short keyword-in-context extracts (as with Google Scholar). The first is primarily indicative, while the second aims to be informative. Extraction of informative text segments from articles is essentially a passage retrieval task – and in some cases, this information may already be available as a natural byproduct of the article ranking process. For example, in algorithms that integrate evidence from multiple spans within an article, those salient spans might form the basis of generating article surrogates.

Even if the primary goal of a system is to directly retrieve relevant passages, the passages must still be couched within the context of the article containing the passages (to provide users with pointers back to the original content). In addition, there will be cases where a passage retrieval algorithm suggests multiple passages extracted from the same article (unless this is explicitly suppressed, which may lead to loss of potentially-important information). To facilitate result presentation, it would be desirable to group passages by the articles that contain them – which essentially involves article ranking.

In other words, the distinction between retrieving passages and retrieving articles becomes blurred when one considers elements of the user interface. Both approaches must grapple with the same issues, thus creating synergies where algorithms specifically developed for one purpose may be useful for the other.

## Conclusion

Experiments in this article with the TREC 2007 genomics track test collection illustrate that there is significant value in searching full-text articles. Given the rapidly growing availability of full text in online digital archives, this is a positive development for scientists who depend on access to the literature for their research. Results show that retrieval at the level of paragraphs within full text is significantly more effective than searching abstracts only. Combining span- and article-level evidence appears to yield the best results. However, much work remains in developing effective full-text retrieval algorithms for the life sciences literature: toward that end, this work presents a first step.

One important issue in moving from searching abstracts to searching full text is that of scalability. Gains in effectiveness come at a cost – algorithms must process significantly more text. Although currently-available tools designed to run on single machines suffice to handle present test collections, it is clear that future systems must distribute computations across multiple machines to cope with ever-growing quantities of text. As illustrated by Ivory, MapReduce provides a convenient framework for distributed text retrieval. The combination of greater effectiveness enabled by full text and greater efficiency enabled by cluster computing paves the way for exciting future developments in information access tools for the life sciences.

## Methods

### Test Collection

Experiments reported in this article were conducted with the test collection from the TREC 2007 genomics track evaluation [26]. A test collection is a standard laboratory tool for evaluating text retrieval systems, which consists of three components:

• a collection – documents on which retrieval is performed,

• a set of information needs – written statements describing the desired information (called "topics"), which are usually provided as queries to the system, and

• relevance judgments – records specifying the documents that should be retrieved in response to each information need (i.e., which documents are relevant to each topic).

The use of test collections to assess the effectiveness of text retrieval algorithms is a well-established methodology in the information retrieval literature, dating back to the Cranfield experiments in the 60's [42]. These tools enable rapid, reproducible experiments in a controlled setting without requiring manual assessment. In modern information retrieval research, test collections are created through large-scale evaluations, such as the Text Retrieval Conferences (TRECs) sponsored by the U.S. National Institute of Standards and Technology (NIST) [43]. TREC is an annual evaluation forum that draws together researchers from around the world to work on shared problems in different "tracks". Over the years, TREC has explored a wide variety of problems ranging from multimedia retrieval to spam detection. The genomics track was dedicated to exploring biomedical text retrieval.

The TREC 2007 genomics track used a collection of 162,259 full-text articles assembled in 2006. These articles came from the electronic distribution of 49 genomics-related journals from Highwire Press. The articles were distributed in HTML, which preserved formatting, structure, table and figure legends, etc. In addition, the organizers gathered MEDLINE records corresponding to each of the full-text articles, which were also made available to participants in the evaluation.

The test collection contains 36 official topics in the form of questions that asked for specific entities such as proteins and drugs – the first five topics are shown in Table 7. Entities of interest are denoted in square brackets and correspond to controlled terminologies from various sources (e.g., MeSH). The topics were created after surveying biologists about recent information needs, and hence can be considered representative for an important group of users who regularly depend on access to the literature.

**Table 7:** Sample topics from the TREC 2007 genomics track.

200	What serum [PROTEINS] change expression in association with high disease activity in lupus?
201	What [MUTATIONS] in the Raf gene are associated with cancer?
202	What [DRUGS] are associated with lysosomal abnormalities in the nervous system?
203	What [CELL OR TISSUE TYPES] express receptor binding sites for vasoactive intestinal peptide (VIP) on their cell surface?
204	What nervous system [CELL OR TISSUE TYPES] synthesize neurosteroids in the brain?
205	What [SIGNS OR SYMPTOMS] of anxiety disorder are related to coronary artery disease?

Relevance judgments consist of lists of legal spans that were determined to contain an answer, based on the opinion of human assessors with significant domain knowledge (Ph.D. in the life sciences). A legal span is a prescribed unit of retrieval that corresponds to a paragraph in the full-text article. The notion of a legal span evolved out of an attempt to standardize system output. Since systems varied in their processing of article text (in terms of segmentation, tokenization, etc.), a prescriptively-defined unit of retrieval made results easier to compare. In total, there are 12.6 million legal spans in the collection; a list of all legal spans was distributed alongside the full-text articles. In the context of this study, an article is considered relevant if it contains at least one relevant legal span.

As a final note, organizers of the TREC 2007 genomics track were unable to gather corresponding MEDLINE records for approximately 1% of the full-text articles in the Highwire collection. This was due to inconsistencies between the document identifiers used by Highwire Press and PMIDs in MEDLINE. According to my analysis, this resulted in the abstract index having 13 fewer relevant articles than the full-text index (out of a total of 2477) for the entire test set of 36 topics. To be consistent, the official relevance judgments were used in all experiments. However, I confirmed that the small number of missing abstracts had no significant impact on results.

### Retrieval Models

Most modern text retrieval systems adopt a "bag of words" model, in which documents are treated as unordered collections of terms. Although such a model ignores the richness and complexity of natural language – disregarding syntax, semantics, and even word order – this simplification has proven to be effective in practice. In one standard formulation of the retrieval problem, a document *d *is represented as a vector *W*_*d *_of term weights *w*_*t*, *d*_, which reflect the importance of each term *t *in the document. A document vector has dimensions |*V*|, the size of the vocabulary in the entire collection. As a matter of convenience, "document" generically refers to the unit of indexing (which may in actuality be an abstract, a paragraph, etc.) A query *q *is represented in the same manner, and the score of a document with respect to the query is computed as follows:

(1) $$\sum_{t\in q}w_{t,d}\cdot w_{t,q}$$

This inner-product formulation is sufficiently general to capture a wide range of retrieval models. Note that since queries are often very short, *w*_*t*, *q *_is generally less important than *w*_*t*, *d*_.

Given a query, a text retrieval system returns a list of documents with respect to a particular retrieval model. In the inner-product formulation, ranking algorithms vary in how term weights are computed. This work explores two different ranking algorithms: the algorithm implemented in the open-source Lucene search engine and Okapi *bm25*.

Lucene is best described as a modified *tf.idf *ranking algorithm. Given a query *q*, the score of a document is computed as the sum of contributions from individual query terms:

$$c\sum_{t\in q}\sqrt{tf}{\left(1+log\frac{N}{n+1}\right)}^{2}\left(\frac{1}{\sqrt{dl}}\right)$$

For each term *t *that appears in the query *q*, *tf *is the term frequency in the document, *N *is the number of documents in the collection, *n *is the number of documents containing *t *(its document frequency), and *dl *is the document length. The first term inside the summation is the *tf *component, the second is the *idf *component, and the third is a length normalization component. On top of a standard inner-product formulation, Lucene introduces *c*, a "coordination factor", defined as the fraction of query terms found in the document. This factor rewards documents that have many matching terms.

Okapi *bm25 *[35,36] models documents as a mixture of two Poisson processes. One process generates so-called *elite *terms, corresponding to those that an author uses in writing about the topic of a particular document. The other process generates *non-elite *terms, corresponding to those that the author uses in passing (i.e., words whose appearance is incidental to the topic of the document). Due to the complexity of parameter estimation for the full two-Poisson formulation, *bm25 *uses an empirically-derived approximation [44]. Given a query *q*, *bm25 *computes the score of a document as the sum of contributions from individual query terms:

$$\sum_{t\in q}\log\left(\frac{N-n+0.5}{n+0.5}\right)\frac{(k_{1}+1)tf}{K+tf}\frac{(k_{3}+1)qtf}{k_{3}+qtf}$$

For each term *t*, *tf *and *qtf *are term frequencies in the document and query, respectively; *N *is the number of documents in the collection; and *n *is the number of documents containing the term. *K*, a length normalization factor, is defined as follows:

$$K=k_{1}\left((1-b)-b\frac{dl}{avdl}\right)$$

where *dl *is the document length and *avdl *is the average length of all documents. The constants *k*_1_, *b*, and *k*_3 _are tunable parameters. In my experiments, I used *k*_1 _= 1.2, *b *= 0.75, and *k*_3 _= 1000, which are typical settings recommended in the literature.

### Ivory: A Toolkit for Distributed Text Retrieval

Ivory is a toolkit for distributed text retrieval being developed at the University of Maryland to explore scalable algorithms [45]. The software was created using Hadoop, which is an open-source Java implementation of the MapReduce programming model [4] originally developed by Google. Ivory supports the large class of retrieval models that can be expressed as an inner product of term weights (see Section 5.2), and currently implements both *bm25 *and the Lucene ranking algorithm (with a special extension to handle the coordination factor).

Certainly, distributed retrieval systems are not new – Web search engines have been in existence for over a decade. However, the exact architectures of these systems are guarded as commercial secrets. Even though outsiders are occasionally offered glimpses into their design [46,47], few details are available about important engineering tradeoffs. On the other hand, most open-source search engines were not specifically designed for multiple machines (or require tedious manual configuration to run on clusters). Although existing tools can easily support text retrieval experiments involving the Highwire collection (and indeed even much larger collections), the growth of available content will inevitably require transition to cluster-based environments. Ivory represents an initial attempt to develop a toolkit for distributed text retrieval using Hadoop; upon suitable maturity, it will be released as open-source software.

MapReduce is an attractive framework for concurrent programming because it frees the software developer from having to explicitly worry about system-level issues such as fault tolerance, synchronization, inter-process communication, scheduling, etc. The abstraction simplifies the design of scalable, distributed algorithms. With the release of Hadoop, an open-source implementation of the MapReduce programming model led by Yahoo, this versatile framework is available to anyone.

MapReduce draws inspiration from higher-order functions in functional programming and builds on the observation that many information processing tasks have the same basic structure: a computation is applied over a large number of records (e.g., Web pages, nodes in a graph) to generate partial results, which are then aggregated in some fashion. In MapReduce, the programmer defines a "mapper" and a "reducer" with the following signatures:

map: (*k*_1_, *v*_1_) → [(*k*_2_, *v*_2_)]

reduce: (*k*_2_, [*v*_2_]) → [(*k*_3_, *v*_3_)]

**Figure 1:**
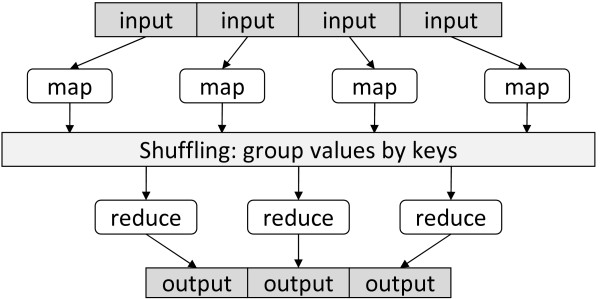
**Illustration of the MapReduce framework: the "mapper" is applied to all input records, which generates results that are aggregated by the "reducer"**. The runtime groups together values by keys.

Key-value pairs form the basic data structure in MapReduce. The mapper is applied to every input key-value pair to generate an arbitrary number of intermediate key-value pairs (I adopt the convention of [...] to denote a list). The reducer is applied to all values associated with the same intermediate key to generate output key-value pairs. This two-stage processing structure is illustrated in Figure 1.

In MapReduce, a programmer need only provide implementations of the mapper and reducer. On top of a distributed file system [48], the runtime transparently handles all other aspects of execution, on clusters ranging from a few to a few thousand nodes. The runtime is responsible for scheduling map and reduce workers, detecting and handling faults, delivering input data, shuffling intermediate results, and gathering final output. The MapReduce abstraction allows many complex algorithms to be expressed concisely.

**Figure 2:**
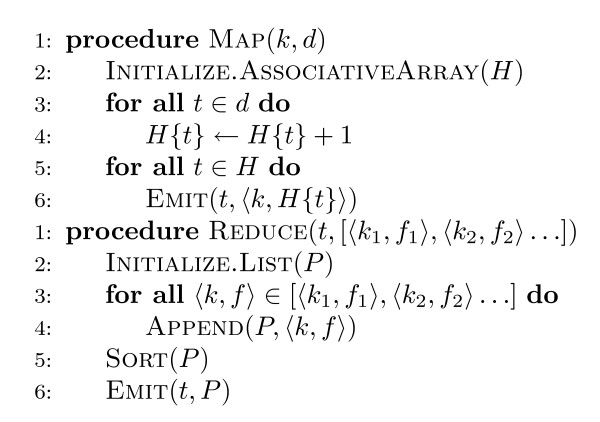
**Pseudo-code of Ivory's indexing algorithm in MapReduce**. The mapper processes each document and emits postings with the associated term as the key. The reducer gathers all postings for each term to create the inverted index.

The pseudo-code for Ivory's indexing algorithm is shown in Figure 2. Like nearly all text retrieval systems, Ivory builds a data structure called an inverted index, which given a term provides access to the list of documents that contain the term. An inverted index consists of postings lists, one associated with each term in the collection. A postings list is comprised of individual postings, each of which represents a (document id, term frequency) pair. This information is used to compute term weights during retrieval.

Input to the indexer consists of document ids (keys) and associated document content (values). In each mapper, the document text is tokenized and stemmed term occurrences are first stored in a histogram *H *(implemented as an associative array). After this histogram has been built, the mapper then iterates over all terms. For each term, a pair consisting of the document id (*k*) and the frequency of the term in the document (*f*) is created. Each pair, denoted by ⟨*k*, *f*⟩ in the pseudo-code, represents an individual posting.

The mapper then emits an intermediate key-value pair with the term as the key and the posting as the value. MapReduce guarantees that all values associated with the same key will be sent to the same reducer; the reducer gathers up all postings, sorts them by descending term frequency, and emits the complete postings list, which is then written out to the distributed file system. The final key-value pairs (terms and associated postings lists) make up the inverted index.

Typically, computing term weights requires information about document lengths. This is straightforwardly expressed as another MapReduce algorithm: each mapper counts up the number of terms in a document and emits the term count as a value with the associated document id as the key. In this case, there is no need for a reducer – document lengths are directly written to disk. The table of document lengths is relatively compact, and is read into memory by each mapper in the retrieval phase.

Retrieval with an inverted index involves fetching postings lists that correspond to query terms, and then scoring documents based on term weights computed from postings information. For real-time applications, this requires low-latency access to the inverted index. However, Hadoop was primarily designed for high-throughput batch computations, not computations for which low latency is desired. Presently, Hadoop does not provide a mechanism for low-latency random access to the distributed file system. To work around this limitation, Ivory's retrieval algorithm was designed for parallel query execution.

**Figure 3:**
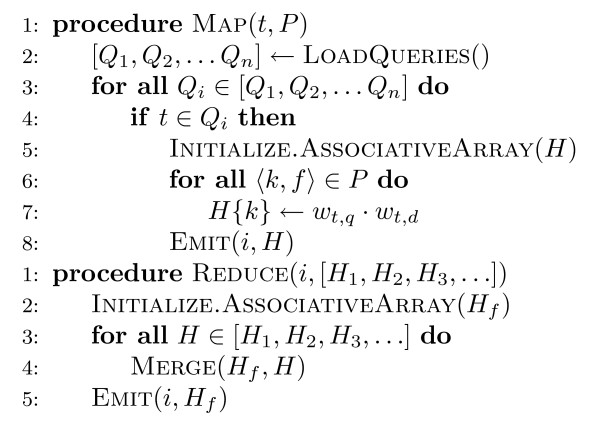
**Pseudo-code of Ivory's retrieval algorithm in MapReduce**. The mapper processes the postings lists in parallel. For each query term, the mapper initializes accumulators to hold partial score contributions from all documents containing the term. The reducer adds up partial scores to produce the final results.

The pseudo-code for Ivory's retrieval algorithm is shown in Figure 3. The input to each mapper is a term *t *(the key) and its associated postings list *P *(the value). The mapper loads up *all *the queries at once and processes each query in turn. If the query does not contain *t*, no action is performed. If the query contains *t*, then the corresponding postings must be traversed to compute the partial contributions to the query-document score. For each posting element, the partial contribution to the score (*w*_*t*, *q*_·*w*_*t*, *d*_) is computed based on the actual ranking algorithm. For example, *bm25 *requires the document frequency of *t *(known by the length of the postings list), term frequency of *t *in the document (stored in the posting), query frequency (loaded by the mapper), document length (stored separately and loaded by the mapper; see above), and average document length (same). With all the necessary components, computing the partial score contribution is simply a matter of arithmetic. Each partial score is stored in an associative array *H*, indexed by the document id *k *– this structure serves the same functionality as accumulators in a traditional retrieval engine. The mapper emits an intermediate key-value pair with the query number *i *as the key and *H *as the value. The result of each mapper is all partial query-document scores associated with term *t *for all queries that contain the term.

In the reduce phase, all associative arrays belonging to the same query are brought together by the runtime. The reducer performs an element-wise sum of all the associative arrays (denoted by the MERGE function in the pseudo-code): this adds up the contributions for each query term across all documents. The final result is an associative array containing scores for all documents that have at least one query term. This structure is then sorted (outside MapReduce by a separate process) to generate the final ranked list for fixed cutoff.

Note that this retrieval algorithm replaces random access of the postings with a parallel scan of all postings – this modification was necessary due to limitations of Hadoop. However, since disk scans are distributed across the entire cluster, it is possible to exploit the aggregate disk bandwidth of all available machines. In processing a set of queries, each postings list is accessed only once – each mapper computes partial score contributions for *all *queries that contain the query term. It should be emphasized that this algorithm is not meant for real-time retrieval applications, but rather is intended for running batch-style experiments in a research context.

## References

[B1] Hunter L, Cohen KB (2006). Biomedical Language Processing: What's Beyond PubMed?. Mol Cell.

[B2] Zweigenbaum P, Demner-Fushman D, Yu H, Cohen KB (2007). New Frontiers In Biomedical Text Mining. Pacific Symposium on Biocomputing 12.

[B3] Zweigenbaum P, Demner-Fushman D, Yu H, Cohen KB (2007). Frontiers of Biomedical Text Mining: Current Progress. Brief Bioinform.

[B4] Dean J, Ghemawat S (2004). MapReduce: Simplified Data Processing on Large Clusters. Proceedings of the 6th Symposium on Operating System Design and Implementation (OSDI 2004), San Francisco, California.

[B5] Gay CW, Kayaalp M, Aronson AR (2005). Semi-Automatic Indexing of Full Text Biomedical Articles. AMIA Annu Symp Proc.

[B6] Yu H, Hatzivassiloglou V, Friedman C, Rzhetsky A, Wilbur WJ (2002). Automatic Extraction of Gene and Protein Synonyms from MEDLINE and Journal Articles. Proc AMIA Symp.

[B7] Seki K, Mostafa J (2007). Discovering Implicit Associations Between Genes and Hereditary Diseases. Pac Symp Biocomput.

[B8] Demner-Fushman D, Hauser S, Thoma G (2005). The Role of Title, Metadata and Abstract in Identifying Clinically Relevant Journal Articles. AMIA Annu Symp Proc.

[B9] Yu H (2006). Towards Answering Biological Questions with Experimental Evidence: Automatically Identifying Text that Summarize Image Content in Full-Text Articles. AMIA Annu Symp Proc.

[B10] Yu H, Lee M (2006). Accessing Bioscience Images from Abstract Sentences. Bioinformatics.

[B11] Shah PK, Perez-Iratxeta C, Bork P, Andrade MA (2003). Information Extraction from Full Text Scientific Articles: Where are the Keywords?. BMC Bioinformatics.

[B12] Schuemie MJ, Weeber M, Schijvenaars BJA, van Mulligen EM, Eijk CC van der, Jelier R, Mons B, Kors JA (2004). Distribution of Information in Biomedical Abstracts and Full-Text Publications. Bioinformatics.

[B13] Wilbur WJ, Smith L, Tanabe L (2007). BioCreative 2. Gene Mention Task. Proceedings of the Second BioCreative Challenge Evaluation Workshop, Madrid, Spain.

[B14] Krallinger M, Leitner F, Valencia A (2007). Assessment of the Second BioCreative PPI Task: Automatic Extraction of Protein-Protein Interactions. Proceedings of the Second BioCreative Challenge Evaluation Workshop, Madrid, Spain.

[B15] Salton G, Buckley C (1991). Automatic Text Structuring and Retrieval – Experiments in Automatic Encyclopedia searching. Proceedings of the 14th Annual International ACM SIGIR Conference on Research and Development in Information Retrieval (SIGIR 1991), Chicago, Illinois.

[B16] Salton G, Allan J, Buckley C (1993). Approaches to Passage Retrieval in Full Text Information Systems. Proceedings of the 16th Annual International ACM SIGIR Conference on Research and Development in Information Retrieval (SIGIR 1993), Pittsburgh, Pennsylvania.

[B17] Hearst MA, Plaunt C (1993). Subtopic Structuring for Full-Length Document Access. Proceedings of the 16th Annual International ACM SIGIR Conference on Research and Development in Information Retrieval (SIGIR 1993), Pittsburgh, Pennsylvania.

[B18] Callan JP (1994). Passage-Level Evidence in Document Retrieval. Proceedings of the 17th Annual International ACM SIGIR Conference on Research and Development in Information Retrieval (SIGIR 1994), Dublin, Ireland.

[B19] Wilkinson R (1994). Effective Retrieval of Structured Documents. Proceedings of the 17th Annual International ACM SIGIR Conference on Research and Development in Information Retrieval (SIGIR 1994), Dublin, Ireland.

[B20] Kaszkiel M, Zobel J (1997). Passage Retrieval Revisited. Proceedings of the 20th Annual International ACM SIGIR Conference on Research and Development in Information Retrieval (SIGIR 1997), Philadelphia, Pennsylvania.

[B21] Clarke C, Cormack G, Tudhope E (2000). Relevance Ranking for One to Three Term Queries. Information Processing and Management.

[B22] Liu X, Croft WB (2002). Passage Retrieval Based on Language Models. Proceedings of the Eleventh International Conference on Information and Knowledge Management (CIKM 2002), McLean, Virginia.

[B23] Tellex S, Katz B, Lin J, Marton G, Fernandes A (2003). Quantitative Evaluation of Passage Retrieval Algorithms for Question Answering. Proceedings of the 26th Annual International ACM SIGIR Conference on Research and Development in Information Retrieval (SIGIR 2003), Toronto, Canada.

[B24] Wang M, Si L (2008). Discriminative Probabilistic Models for Passage Based Retrieval. In Proceedings of the 31st Annual International ACM SIGIR Conference on Research and Development in Information Retrieval (SIGIR 2008), Singapore.

[B25] Lalmas M, Tombros A (2007). INEX 2002–2006: Understanding XML Retrieval Evaluation. Digital Libraries: Research and Development – First International DELOS Conference, Revised Selected Papers, Pisa, Italy.

[B26] Hersh WR, Cohen A, Ruslen L, Roberts P (2007). TREC 2007 Genomics Track Overview. Proceedings of the Sixteenth Text REtrieval Conference (TREC 2007), Gaithersburg, Maryland.

[B27] Trotman A (2005). Wanted: Element Retrieval Users. Proceedings of the INEX 2005 Workshop on Element Retrieval Methodology, Glasgow, Scotland.

[B28] Larsen B, Tombros A, Malik S (2006). Is XML Retrieval Meaningful to Users? Searcher Preferences for Full Documents vs. Elements. Proceedings of the 29th Annual International ACM SIGIR Conference on Research and Development in Information Retrieval (SIGIR 2006), Seattle, Washington.

[B29] Salton G, Wong Y, Yang CS (1975). A Vector Space Model for Automatic Indexing. Communications of the ACM.

[B30] Ponte JM, Croft WB (1998). A Language Modeling Approach to Information Retrieval. Proceedings of the 21st Annual International ACM SIGIR Conference on Research and Development in Information Retrieval (SIGIR 1998), Melbourne, Australia.

[B31] Metzler D, Croft WB (2004). Combining the Language Model and Inference Network Approaches to Retrieval. Information Processing and Management.

[B32] Rafkind B, Lee M, Chang SF, Yu H (2006). Exploring Text and Image Features to Classify Images in Bioscience Literature. Proceedings of the HLT/NAACL 2006 Workshop on Biomedical Natural Language Processing (BioNLP'06), New York, New York.

[B33] Shatkay H, Chen N, Blostein D (2006). Integrating Image Data into Biomedical Text Categorization. Bioinformatics.

[B34] Kou Z, Cohen WW, Murphy RF (2007). A Stacked Graphical Model for Associating Sub-Images with Sub-Captions. Pacific Symposium on Biocomputing 12.

[B35] Robertson SE, Walker S, Hancock-Beaulieu M, Gatford M, Payne A (1995). Okapi at TREC-4. Proceedings of the Fourth Text REtrieval Conference (TREC-4), Gaithersburg, Maryland.

[B36] Sparck Jones K, Walker S, Robertson SE (2000). A Probabilistic Model of Information Retrieval: Development and Comparative Experiments (Parts 1 and 2). Information Processing and Management.

[B37] Singhal A, Buckley C, Mitra M (1996). Pivoted Document Length Normalization. Proceedings of the 19th Annual International ACM SIGIR Conference on Research and Development in Information Retrieval (SIGIR 1996), Zürich, Switzerland.

[B38] Kamps J, de Rijke M, Sigurbjörnsson B (2004). Length Normalization in XML Retrieval. Proceedings of the 27th Annual International ACM SIGIR Conference on Research and Development in Information Retrieval (SIGIR 2004), Sheffield, United Kingdom.

[B39] Ogilvie P, Callan J (2005). Parameter Estimation for a Simple Hierarchical Generative Model for XML Retrieval. Proceedings of the 2005 Initiative for the Evaluation of XML Retrieval Workshop (INEX 2005), Dagstuhl, Germany.

[B40] Sigurbjörnsson B, Kamps J, de Rijke M (2003). An Element-based Approach to XML Retrieval. Proceedings of the 2003 Initiative for the Evaluation of XML Retrieval Workshop (INEX 2005), Dagstuhl, Germany.

[B41] Tbahriti I, Chichester C, Lisacek F, Ruch P (2006). Using Argumentation to Retrieve Articles with Similar Citations: An Inquiry into Improving Related Articles Search in the MEDLINE Digital Library. Int J Med Inform.

[B42] Cleverdon CW, Mills J, Keen EM (1968). Factors Determining the Performance of Indexing Systems.

[B43] Harman DK, Voorhees EM, Harman DK (2005). The TREC Test Collections. TREC: Experiment and Evaluation in Information Retrieval.

[B44] Robertson SE, Walker S (1994). Some Simple Effective Approximations to the 2-Poisson Model for Probabilistic Weighted Retrieval. Proceedings of the 17th Annual International ACM SIGIR Conference on Research and Development in Information Retrieval (SIGIR 1994), Dublin, Ireland.

[B45] Elsayed T, Lin J, Oard D (2008). Pairwise Document Similarity in Large Collections with MapReduce. Proceedings of the 46th Annual Meeting of the Association for Computational Linguistics (ACL 2008), Companion Volume, Columbus, Ohio.

[B46] Brin S, Page L (1998). The Anatomy of a Large-Scale Hypertextual Web Search Engine. Proceedings of the Seventh International World Wide Web Conference (WWW 7), Brisbane, Australia.

[B47] Barroso LA, Dean J, Hölzle U (2003). Web Search for a Planet: The Google Cluster Architecture. IEEE Micro.

[B48] Ghemawat S, Gobioff H, Leung ST (2003). The Google File System. Proceedings of the 19th ACM Symposium on Operating Systems Principles (SOSP 2003), Bolton Landing, New York.

